# Assisted mothers and burnout symptoms: why help matters

**DOI:** 10.1192/j.eurpsy.2026.11840

**Published:** 2026-07-31

**Authors:** E. Polonetskaya, E. Pervichko

**Affiliations:** Faculty of Psychology, Lomonosov Moscow State University, Moscow, Russian Federation

## Abstract

**Introduction:**

Apart from the inherent psychological workload, motherhood increases the volume of most basic household chores and requires extensive engagement in caring for one’s child. Nowadays, as nuclear families become more widespread, mothers are commonly left to face this struggle alone.

**Objectives:**

Our research was aimed at studying the associations between the amount of help extended to mothers (with household chores and/or with childcare) and maternal burnout during the first years of motherhood.

**Methods:**

A maternal burnout questionnaire in Russian (the Maternal Burnout Assessment Tool, MBAT), being an adaptation of the Burnout Assessment Tool to the motherhood context, was used to assess burnout tendencies in mothers. The sample consisted of 792 Russian-speaking mothers with children up to seven years of age. Single-factor analysis of variance was used for data analysis (Kruskal-Wallis test).

**Results:**

Our research has shown that 45% of mothers receive an insignificant amount of help with childcare (where the mother would be left on her own for at least several hours a month), while 82% of mothers receive little to no assistance with household chores. The relevant mean burnout scores and p for the relevant single-factor analysis of variance are shown in Table 1 and Table 2 below, statistically significant differences according to the Dwass-Steel-Critchlow-Fligner paiwise comparison are marked with asterisks. No statistically significant differences in the burnout symptoms not shown below (such as cognitive impairment) were discovered. identification of most vulnerable groups

**Table 1.** Table 1. Long description.

Assistance with childcare	No assistance	Several hours a month	Several hours a week	Several hours a day	The mother is not the primary caregiver	*p*
**Burnout**	2.64	2.71*	2.60	2.51*	2.80	.043
**Exhaustion**	3.02	3.10*	2.93	2.83*	3.11	.028
**Distancing**	2.12*	2.16**	2.07	1.87* **	2.29	.001

**Table 2.** Table 2. Long description.

Assistance with household chores	Almost no assistance	Limited assistance	Assistance with half of the chores	Assistance with most of the chores	*p*
**Burnout**	2.73*	2.53*	2.55	2.65	.003
**Exhaustion**	3.12*	2.86*	2.90	2.96	.001
**Distancing**	2.19*	1.96*	1.99	2.15	.004
**Cognitive impairment**	2.37*	2.22*	2.21	2.44	.029

**Conclusions:**

Assistance with childcare and/or household chores provided to a mother is associated with a more moderate manifestation of the symptoms of maternal burnout. It might be argued that the most burned-out mothers are those actively seeking external assistance with childcare. This should be considered on both a family and public level.

**Disclosure of Interest:**

None Declared

## Long descriptions

### Table 1. Long description

The table consists of seven columns and four rows.

Columns from left to right are:

1. Assistance with childcare (Row labels)

2. No assistance

3. Several hours a month

4. Several hours a week

5. Several hours a day

6. The mother is not the primary caregiver

7. p (significance value)

Data rows:

- Burnout: No assistance (2.64), Several hours a month (2.71*), Several hours a week (2.60), Several hours a day (2.51*), Mother not primary (2.80), p value (.043).

- Exhaustion: No assistance (3.02), Several hours a month (3.10*), Several hours a week (2.93), Several hours a day (2.83*), Mother not primary (3.11), p value (.028).

- Distancing: No assistance (2.12*), Several hours a month (2.16**), Several hours a week (2.07), Several hours a day (1.87* **), Mother not primary (2.29), p value (.001).

Asterisks indicate statistical significance levels as noted in the data points.

Navigate back to Table 1..

### Table 2. Long description

The table consists of six columns and five rows. The columns are labeled from left to right as: Assistance with household chores, Almost no assistance, Limited assistance, Assistance with half of the chores, Assistance with most of the chores, and p-value.

* Row 1 (Burnout): Almost no assistance 2.73 asterisk, Limited assistance 2.53 asterisk, Half chores 2.55, Most chores 2.65, p-value .003.

* Row 2 (Exhaustion): Almost no assistance 3.12 asterisk, Limited assistance 2.86 asterisk, Half chores 2.90, Most chores 2.96, p-value .001.

* Row 3 (Distancing): Almost no assistance 2.19 asterisk, Limited assistance 1.96 asterisk, Half chores 1.99, Most chores 2.15, p-value .004.

* Row 4 (Cognitive impairment): Almost no assistance 2.37 asterisk, Limited assistance 2.22 asterisk, Half chores 2.21, Most chores 2.44, p-value .029.

Navigate back to Table 2..

